# Screening for pregestational and gestational diabetes in pregnancy: a survey of obstetrical centers in the northern part of Belgium

**DOI:** 10.1186/1758-5996-5-66

**Published:** 2013-11-11

**Authors:** Katrien Benhalima, Paul Van Crombrugge, Roland Devlieger, Johan Verhaeghe, Ann Verhaegen, Luc De Catte, Chantal Mathieu

**Affiliations:** 1 Department of Endocrinology, UZ Gasthuisberg, KU Leuven, Leuven, Belgium; 2 Department of Endocrinology, OLV ziekenhuis Aalst-Asse-Ninove, Aalst, Belgium; 3 Department of Obstetrics & Gynecology, UZ Gasthuisberg, KU Leuven, Leuven, Belgium; 4 Chair of the professional working group of the Flemish Diabetes Association (VDV), Gent, Belgium; 5 Chair of the obstetrical working group of the Flemish Association of Obstetrics & Gynecology (VVOG), Sint-Niklaas, Belgium

**Keywords:** Survey, Screening, Pregestational diabetes, Gestational diabetes, Practices

## Abstract

**Background:**

There is lack of consensus concerning the best screening strategy for gestational diabetes (GDM). The aim of our survey was therefore to investigate attitudes and practices of all obstetrical centers in the northern part of Belgium regarding screening for pregestational diabetes in early pregnancy and screening for GDM. We also aimed to identify the penetrance of the ‘International Association of Diabetes in Pregnancy Study Groups’ (IADPSG) screening strategy for GDM.

**Methods:**

The survey was conducted from May 2012 till January 2013. The survey was distributed to every obstetrical center in the northern part of Belgium by email and/or mail with reminders by phone and personal contact.

**Results:**

From the 65 obstetrical centers, 69% responded. Of all centers, 27% had a structured database on the number of women with GDM. Of all centers, 82% screened for pregestational diabetes in early pregnancy and 56% of centers screened for GDM before 24 weeks. Screening before 24 weeks was mostly based on risk factors. Screening for GDM after 24 weeks, was done universally in 87% of centers. The mean estimated prevalence of GDM was 7 ± 5%. The most commonly used screening strategy was a two-step approach with a glucose challenge test (GCT) and 100 g oral glucose tolerance test (OGTT), used by 56% of centers, with 23 centers using the Carpenter & Coustan criteria. The 75 g OGTT with the IADPSG criteria was used by 33% of centers but 4 of these centers still used a GCT before proceeding to the full OGTT.

**Conclusions:**

This survey demonstrates that in the northern part of Belgium, there still is a large variation in screening strategy for pregestational diabetes in early pregnancy and GDM. Only 25% of centers have already implemented the one-step IADPSG screening strategy.

## Background

Besides the worldwide increase of type 2 diabetes (T2DM) in younger adults, the maternal age at first pregnancy also increases in the western world. The timely detection of dysglycemia very early in pregnancy is therefore essential as these women have an increased risk for congenital anomalies [1]. The timely detection of gestational diabetes (GDM) is important since the risk for fetal overgrowth and the risk for the development of T2DM postpartum [2,3]. The ‘International Association of Diabetes and Pregnancy Study Groups’ (IADPSG) consensus recommends now an universal screening with the 2-h 75 g oral glucose tolerance test (OGTT) from 24-28 weeks of gestation using more stringent diagnostic criteria. Moreover, one abnormal value is now enough for the diagnosis of GDM [4]. Internationally there still is a lot of controversy concerning the IADPSG recommendation for screening for GDM. In most populations, the implementation of the IADPSG screening strategy will probably lead to an important increase in the number of women labeled and treated as GDM. The American Diabetes Association (ADA) has adopted the IADPSG recommendation since December 2010, while the American College of Obstetricians and Gynecologists advises to continue with the two-step screening strategy (universal screening with a 50 g glucose challenge test (GCT), followed by a 3-h 100 g OGTT only when the GCT is abnormal) [5,6]. In March 2013 an independent expert panel, appointed by the National Institute of Health, recommended that the two-step approach be continued [7]. The panel is particularly concerned that the adoption of the IADPSG criteria would increase the prevalence of GDM, and the corresponding costs and interventions, without clear demonstration of improvements in the most clinically important health and patient-centered outcomes.

The discrepancy in recommendations is also apparent in Belgium. In 2012 a Flemish consensus between endocrinologists, obstetricians and primary care physicians decided that at this moment there is not enough evidence to already implement the IADPSG screening strategy for GDM and therefore recommended to continue with the two-step screening strategy [8]. In contrast, a recent consensus of ‘le Groupement des Gynécologues Obstétriciens de Langue Française de Belgique’ was to adopt the proposed IADPSG screening strategy for GDM [9].

Due to the lack of consensus on the best screening strategy for GDM both internationally and nationally, the aim of our survey was to investigate attitudes and practices of all obstetrical centers in the northern part of Belgium regarding screening for pregestational diabetes in early pregnancy and screening for GDM. We also aimed to identify the penetrance of the IADPSG screening strategy for GDM.

## Methods

An anonymous survey was designed to evaluate attitudes and practices concerning screening for pregestational diabetes in early pregnancy and screening for GDM [Additional file 1]. The current study was in compliance with the Helsinki Declaration.

The initial segment of the survey included specific questions about the general characteristics of the obstetrical center and practice details. The next segment surveyed provider attitudes on screening for GDM. The following questions concerned the information included in the protocol on GDM, if and how women were screened for pregestational diabetes at first prenatal visit and how screening was performed for GDM before and after 24 weeks of pregnancy. Providers could indicate that they used more than one type of screening test if necessary. The ultimate segment dealt with questions on the follow-up strategy at delivery and postpartum to screen for T2DM.

The survey was conducted from May 2012 till January 2013, before the publication of the Flemish consensus on screening for GDM. The survey was distributed to obstetricians at conferences in the northern part of Belgium (Flanders) or distributed to every obstetrical center by email and/or mail. If the survey was not returned within two months, obstetricians were reminded by phone and/or personal contact. There are 65 obstetrical centers in Flanders. The aim was to obtain one survey for every obstetrical center.

The background prevalence of T2DM in Belgium is 7.0% compared to a mean prevalence of T2DM in Europe of 8.3% [10]. Belgium has a population of nearly 11 million of which 12% are from an ethnic minority background. 6.3 million of all Belgians live in Flanders. In the general adult population 28% of women are overweight and 13% are obese [11]. At the start of this century, Flanders had the lowest rate of mothers aged 35 or more (10.9%) and one of the lowest rates of teenage pregnancies (2.2%) among 16 regions in Western Europe [12].

Statistical analyses were performed using SPSS 19.0. Continuous variables (normally distributed) are expressed as mean (SD) or expressed as median if not normally distributed. Non categorical data expressed as percentage. To compare variables between the different groups independent samples T-tests was used for normally distributed continuous variables and chi-squared tests for categorical variables.

## Results

Of all the 65 centers who received the survey, 45 completed the survey, leading to a response rate of 69%. Responders included 42 obstetricians and 3 endocrinologists. The provinces Limburg, Flemish Brabant and East Flanders had the highest response rate (resp. 88%, 71% and 69%), followed by the province of West Flanders (60%) and Antwerp (55%).

### General characteristics

Of all responders, 7% worked in an university hospital, 27% worked in a non-university training hospital and 66% worked in a community based hospital. The mean number of obstetricians per center was 6 (range 3-16). The median number of deliveries per year per center was 900 (range 400-2700). Of all centers, 27% (12) had a database with the number of GDM women registered. The estimated mean prevalence of GDM was 7 ± 5% but with a very large variation (1-20%).

### Attitudes of providers

All responders but one believed that it was beneficial to screen for GDM. Moreover, a very large majority (91% of responders) considered that screening for GDM was well organized in their center. Only four responders considered that screening for GDM was not well organized in their center due to the absence of a protocol (1), an incomplete protocol (2) or logistical problems (1). Of all responders, 38% (17) considered that the estimated risk for women with previous GDM to develop T2DM in the next 10 years after the index pregnancy, is lower than 30%.

### Protocol for GDM

Of all centers, 73% (33) had a written protocol regarding the policy for GDM. Information on screening for pregestational diabetes at first prenatal visit was present in 33% (15) of centers and information on the obstetrical follow up was present in 44% (20) of centers. Information on the delivery modalities was available in 56% (25) of centers and information on the follow-up strategy to screen for diabetes after delivery was present in 51% (23) of centers. In all centers both obstetricians and endocrinologists were involved in the development of the protocol. Pediatricians were involved in the development of the protocol in 38% (17) of centers, primary care physicians in only 7% (3) of centers, midwifes in 33% (15) of centers, diabetes nurses in 56% (25) of centers and dieticians in 47% (21) of centers.

### Screening for pregestational diabetes in early pregnancy

Of all centers, 82% (37) regularly screened for pregestational diabetes in early pregnancy. A risk profile was assessed by 46% (17) of centers before a screening test was used. The most commonly used screening tests were the measurement of a fasting plasma glucose (FPG) (35%) or a random glycaemia (35%) [Table 1]. The estimated number of women screened for pregestational diabetes in early pregnancy was 65% (± 31%) but with a very wide variation between centers (5-99%). The estimated number of women who attended a preconceptional clinic was 21% (± 22%).

**Table 1:** An overview of the screening tests used to screen for pregestational diabetes in early pregnancy, for GDM before 24 weeks of pregnancy and for GDM ≥ 24 weeks of pregnancy

**Screening tests used**	**Pregestational diabetes (n = 37)**	**GDM < 24 weeks (n = 25)**	**GDM ≥ 24 weeks (n = 45)**
FPG	35% (13)	32% (8)	0
HbA1c	14% (5)	4% (1)	2% (1)
Random glycaemia	35% (13)	28% (7)	0
Glycosuria	30% (11)	4% (1)	0
Combination of tests	14% (5)	52% (13)	9% (4)
Combination of GCT and OGTT			
≥ 130 mg/dl	0	8% (2)	16% (7)
≥ 140 mg/dl	0	40% (10)	64% (29)
One-step OGTT			
75 g	0	24% (6)	27% (12)
100 g	0	12% (3)	0

GDM: gestational diabetes; FPG: fasting plasma glucose; GCT: 50 g glucose challenge test; OGTT: oral glucose tolerance test.

### Screening for GDM before 24 weeks of pregnancy

Of all centers, 56% (25) screened for GDM before 24 weeks of pregnancy. The estimated number of women screened for GDM before 24 weeks of pregnancy was 30% (± 33%). Screening based on risk factors was performed by 67% (16) of centers. The most common used risk factors to screen for GDM were a family history of diabetes (75%), a history of GDM (71%), being overweight or obese (68%), a history of a macrosomic baby (29%) and a personal history of PCOS (14%). The most common used screening tests were a GCT with a threshold ≥ 140 mg/dl (40%), a FPG (32%), a random glycaemia (28%) and a one-step screening approach with a 75g OGTT (24%) [Table 1]. In more than half of the centers, providers indicated that different screening tests could be used. When a 75 g OGTT was used, the most common diagnostic criteria used were the IADPSG criteria (28%) [Table 2]. Of the 7 centers who used the IADPSG criteria, 6 centers did this in a one-step approach. When the 100 g OGTT was used, the most common diagnostic criteria used were the Carpenter & Coustan criteria (48%). Three of the 12 centers who used the Carpenter & Coustan criteria with the 100 g OGTT, did this as a one-step approach without GCT.

**Table 2:** An overview of the diagnostic criteria of the OGTT used for GDM before 24 weeks of pregnancy and for GDM ≥ 24 weeks of pregnancy

**Diagnostic criteria**	**GDM < 24 weeks**	**GDM ≥ 24 weeks**
	**(n = 25)**	**(n = 45)**
75 g OGTT		
Carpenter & Coustan	20% (5)	9% (4)
WHO	0	2% (1)
IADPSG	28% (7)	33% (15)
100 g OGTT		
Carpenter & Coustan	48% (12)	52% (23)
NDDG	4% (1)	4% (2)

GDM: gestational diabetes; OGTT: oral glucose tolerance test; WHO: World Health Association; IADPSG: International Association of Diabetes in Pregnancy Study Groups; NDDG: National Diabetes Data Group.

### Screening for GDM ≥ 24 weeks of pregnancy

All centers screened for GDM ≥ 24 weeks of pregnancy and 87% (39) screened universally for GDM. Screening for GDM was mostly performed between 24 weeks (range 20-28) and 29 weeks (range 26-34). The most common used screening tests were a GCT with a threshold ≥ 140 mg/dl (64%), followed by a one-step approach with the 75 g OGTT (27%) and a GCT with a threshold ≥ 130 mg/dl (16%) [Table 1]. The most commonly used screening strategy was a two-step approach with a GCT and 100 g OGTT, used by 56% of centers (25). The Carpenter & Coustan criteria were the most commonly used diagnostic criteria in a two-step approach [Table 2]. Two of the three university hospitals who participated in the survey, also used the two-step approach with the Carpenter & Coustan criteria. When a 75 g OGTT was used, the most commonly used diagnostic criteria were the IADPSG criteria (33%). However, only 11 centers (24%) used the IADPSG criteria as an one-step strategy since four centers still used a GCT (three centers with a threshold ≥ 140 mg/dl and one center with a threshold ≥ 130 mg/dl) before proceeding to the full OGTT. Estimated prevalences of GDM were not different between the centers who used a one-step approach compared to the centers who used a two-step approach (estimated mean prevalence of 8% ± 2 vs. 7% ± 1, p = 0.519).

### Follow up at delivery and postpartum

The protocol concerning the policy at delivery, included information on monitoring of glycaemia at delivery in 84% of centers and information on the need of an insulin sliding scale in 79% of centers. The protocol also included information on the need for an induction in 56% of centers and it included information on the need for a caesarean section in 23% of centers. Information concerning neonatal care on the monitoring of blood glucose in newborns was present in 86% of centers and information on the need for admission on the neonatal intensive care unit was available in 40% of centers. A protocol concerning the long term policy for the evaluation of the risk of women with previous GDM to develop T2DM after delivery, was available in 66% of centers. The most common follow up strategy was registration of women with GDM in the Flemish project ‘Zoet Zwanger’ (‘Sweet Pregnancy’) in 78% (35) of centers. This project is an initiative of the Flemish Diabetes Association and supported by the Flemish government, whereby women receive yearly remainders to have the FPG checked by their general practitioner [13]. Monitoring of blood glucose in hospital after the delivery was done by 47% (21) centers. An universal 75 g OGTT postpartum was done in 33% (15) of centers and a 75 g OGTT only in insulin treated women was done in 22% (10) of centers. Measurement of FPG was done in 13% (6) of centers, while the measurement of Hba1c was only done in 9% (4) of centers and self-monitoring of blood glucose at home was done in only 7% (3) of centers. Of all OGTTs postpartum, the 75 g OGTT was performed less than 6 weeks in 7% of centers, between 6-12 weeks postpartum in 71% of centers and more than 12 weeks postpartum in 19% of centers. Women commonly received advice on diet and weight (91%), on physical activity (86%) and on screening for T2DM (81%). Advice on the need for preconception control was given in 52% of centers and advice on the preferred choice of contraceptives was given in 17% of centers.

## Discussion

This is the first large survey evaluating current practices on screening for pregestational diabetes in early pregnancy and GDM in the northern part of Belgium. The debate on screening for GDM is clearly of concern to the obstetricians, as reflected by the good response rate of nearly 70% of this survey. The survey is also representative for the whole region since there was a response rate of more than 50% in every province.

Responders generally believed that it was beneficial to screen for GDM and that screening for GDM was well organized in their center. Despite this, our survey demonstrates that there is a large variation between the different centers in the northern part of Belgium concerning the strategy used for screening for GDM. More than half of all centers screened for GDM before 24 weeks of pregnancy, mostly based on risk factors. However, many providers indicated that they did not have one particular screening test of choice and that different screening tests could be used. The IADPSG consensus recommends now that a FPG ≥ 92 mg/dl in early pregnancy can be classified as GDM [4]. This is however very debated. A recent evaluation of the FPG in the first prenatal visit to diagnose GDM in China showed that a FPG between 110-125 mg/dl was a much better predictor of the development of GDM and that for their population at least, a FPG ≥ 92 mg/dl at first prenatal visit could not be supported as the criterion for diagnosis of GDM [14].

To screen for GDM ≥ 24 weeks, more than half of all centers used the two-step approach with the 100 g OGTT and Carpenter & Coustan criteria. The most commonly used cutoff for the GCT in this survey was a threshold of ≥140 mg/dl. This has shown to identify about 80% of women with GDM [15]. The yield of the GCT is further increased to 90% by using a cutoff of ≥130 mg/dl but this will inevitably lead to more negative OGTTs. Nearly one-fourth of all centers implemented the one-step IADPSG strategy for GDM. However, 4 of the 15 centers using the IADPSG criteria, used it in a two-step screening strategy with a GCT. This is probably applied as a practical solution since in a center with more than 1000 deliveries per year, a one-step approach would lead to an important increase in workload with the need of at least 3-4 OGTTs daily. The use of a GCT as a universal screening tool in a two-step approach with the use of the IADPSG criteria, is however not yet validated. A contributing factor to this large variation in practices is probably the differing recommendations by both international and local scientific professional organizations. Various large surveys completed by responders from many different countries highlight the strong variability that exists internationally in screening, diagnosis and management of women with GDM [16-18]. Comparison between countries is very difficult due to the different diagnostic strategies and subpopulations. A recent extensive review on the current screening practices in Europe shows that screening practice and policy is very inconsistent across Europe, hampered by lack of consensus and poor clinician awareness of GDM and its diagnosis [19]. Our survey now shows that practices are also very variable in one region.

Nearly one third of all centers had a structured database on the number of women with GDM but there are currently no accurate data on the prevalence of GDM in Belgium. The Flemish birth register data of 2011 showed a prevalence of 2.9% of mothers with diabetes, including both pregestational diabetes as well as GDM [20]. A recent retrospective analysis of the GDM prevalence in the university hospital of Leuven, showed a GDM prevalence of 3.3% using the two-step approach [21]. In this survey the estimated mean prevalence of GDM was 7% but this was mostly based on crude estimations. This probably explains the very large variations in GDM prevalence between the different centers and the absence of a significant difference in reported GDM prevalence between centers using the one-step approach with the IADPSG criteria compared to centers using a two-step approach. Many studies have shown an important increase in GDM prevalence when the IADPSG recommendations are implemented [22,23].

In Flanders the mean maternal age at first pregnancy is now 28 years with 2.3% of women being ≥ 40 years old at first pregnancy [20]. Moreover, the prevalence of overweight and obese women continues to increase. The timely detection of dysglycemia very early in pregnancy is therefore essential. Our survey shows that screening for pregestational diabetes in early pregnancy was often done based on risk factors and by using a FPG or random glycaemia as screening tests. It is generally considered that there is insufficient evidence to recommend one test over the other. The ADA now also recommends to screen for undiagnosed T2DM at the first prenatal visit in those with risk factors using standard diagnostic criteria for a non-pregnant population but does not endorse the recommendation of IADPSG to classify a FPG ≥ 92 mg/dl in early pregnancy as GDM [5]. The recent Flemish consensus recommends to screen with a FPG since this has the advantage that it is easy to perform at a low cost [8]. A FPG has also been shown to diagnose more people than Hba1c, which in turn is more sensitive than a random glycaemia [24]. Measurement of Hba1c on the other hand does not require the patient to fast, which can be especially challenging for pregnant women. However, the reference range for Hba1c is lower during pregnancy due to a reduced FPG and changes in erythrocyte turnover. Compared to non-pregnant women, Hba1c is decreased up to 0.5%, especially in the first and second trimesters [25].

The best postpartum screening strategy for glucose intolerance among women with a history of GDM is still debated. The ADA now recommends to screen women with a history of GDM at 6-12 weeks postpartum using the 2-h 75 g OGTT and non-pregnancy diagnostic criteria but this is mostly based on expert consensus or clinical experience [5]. The most common follow up strategy in our survey, was registration of women with GDM in the Flemish project ‘Zoet Zwanger’ (‘Sweet Pregnancy’). An 75 g OGTT postpartum (universally or only in insulin treated women) was performed in more than half of all centers. The estimated risk for women with previous GDM to develop T2DM in the next 10 years after the index pregnancy, was often underestimated in our survey. This highlights the need for stronger awareness among obstetricians for the risk of women with GDM to develop T2DM after pregnancy.

Strengths of this survey are the good response rate and the detailed questions on screening for pregestational diabetes in early pregnancy, on screening for GDM both before and ≥ 24 weeks of pregnancy and on the follow-up strategy postpartum. Since the aim was to obtain one survey per obstetrical center, it cannot be excluded that within one center different screening strategies are used by different providers. However, we feel that this survey is representative since most centers had a written protocol regarding the policy for GDM.

In conclusion, despite the fact that responders generally believed that it was beneficial to screen for GDM, this survey demonstrates that there is a large variation between the different centers in the northern part of Belgium concerning the strategy used for screening for pregestational diabetes in early pregnancy and screening for GDM. Only one-fourth of centers have implemented the one-step IADPSG screening strategy. A contributing factor to this large variation in practices is probably the differing recommendations by both international and local scientific professional organizations. More research is necessary to investigate the most appropriate screening strategy for pregestational diabetes in early pregnancy and to search for the most cost effective screening strategy for GDM in our population. The development of an uniform and cost effective screening strategy in Belgium, will allow more women during pregnancy to timely receive treatment with glucose-lowering therapy to improve obstetrical outcomes and this will also allow for a more timely detection of T2DM after pregnancy.

## Abbreviations

GDM: Gestational diabetes; IADPSG: International association of diabetes in pregnancy study groups; GCT: Glucose challenge test; OGTT: Oral glucose tolerance test; T2DM: Type 2 diabetes; FPG: Fasting plasma glucose.

## Competing interests

There are no competing interests of this manuscript for neither of the authors.

## Authors’ contributions

KB, PVC and CM designed the survey. KB, RD and JV were involved in the analyses of the survey. KB, AV and LD were involved in the design and distribution of the survey to all the centers. All authors read and approved the final manuscript.

## Supplementary Material

Additional file 1 A copy of the whole survey of the practices in obstetrical centers in the northern part of Belgium concerning screening for pregestational diabetes in early pregnancy and screening for gestational diabetes (GDM). Click here for file
