# Supplementary material for: Screening for pregestational and gestational diabetes in pregnancy: a survey of obstetrical centers in the northern part of Belgium
Source: Diabetol Metab Syndr. 2013 Nov 11;5:66. doi: 10.1186/1758-5996-5-66 (PMC3833269; doi:10.1186/1758-5996-5-66)
Supplement: Additional file 1 — A copy of the whole survey of the practices in obstetrical centers in the northern part of Belgium concerning screening for pregestational diabetes in early pregnancy and screening for gestational diabetes (GDM). [file 1758-5996-5-66-S1.docx]

A copy of the whole survey of the practices in obstetrical centers in the northern part of Belgium concerning screening for pregestational diabetes in early pregnancy and screening for gestational diabetes (GDM)

**General data**

*Center: □ university hospital

□ non-university training hospital

□ community based hospital

□ other: …

*The number of obstetricians working in the hospital: ….

| per obstetrician | 1 | 2 | 3 | 4 | 5 | 6 | 7 | 8 | 9 | 10 |
| --- | --- | --- | --- | --- | --- | --- | --- | --- | --- | --- |
| gender |  |  |  |  |  |  |  |  |  |  |
| age |  |  |  |  |  |  |  |  |  |  |

*The mean number of deliveries per year in your center:

*Does your center have a database with the number of GDM women registered?

□ yes □ no

* What percentage of women have on average GDM in your center?

□ I know… % □ I estimate …% □ I do not know

**General questions**

* According to you, what percentage of women with previous GDM will develop type 2 diabetes within 10 years after the index pregnancy? … %

□ I do not know

* Do you believe that it is beneficial to screen for GDM? □ yes □ no

If not: why?...

* Do you consider that screening for GDM is well organized in your center? □ yes □ no

If not: why?...

**Protocol concerning the policy on gestational diabetes**

*Does you center have a written protocol concerning the policy on GDM?

□ yes □ no

*The protocol contains information on:

□ screening for pregestational diabetes at first prenatal visit

□ screening for GDM

□ the obstetrical follow up

□ the delivery modalities

□ the follow-up strategy to screen for diabetes after delivery

* Which of the following physicians and/or paramedics were involved in the development of the protocol?

□obstetrician

□endocrinologist

□pediatrician

□family physician

□midwife

□diabetes specialist nurse

□ dietician

**Screening at first prenatal visit**

* What is the policy in your center concerning screening for pregestational diabetes at first prenatal visit?

□ nothing

□ assessment of the risk profile and if necessary followed by further testing

□ measurement of a fasting plasma glucose

□ measurement of Hba1c

□ measurement of a random glycaemie

□ measurement of glucosuria

*When you use an assessment of the risk profile, which risk factors do you use? …

*When you screen in early pregnancy, what is the estimated percentage of pregnant women who receive screening?

□ I know… % □ I estimate …% □ I do not know

*What is the estimated percentage of women who attend a preconception clinic?

□ I know… % □ I estimate …% □ I do not know

**Screening for gestational diabetes before 24 weeks of pregnancy**

*Do you screen for GDM before 24 weeks of pregnancy? □ yes □ no

If you screen, what is the estimated percentage of women who receive screening before 24 weeks of pregnancy?

□ I know… % □ I estimate …% □ I do not know

* If you screen for GDM before 24 weeks of pregnancy, is this based on:

□ risk factors □ universally

* If you screen based on risk factors, which risk factors do you use? …

*Which of the following screening tests do you use:

□ measurement of a fasting plasma glucose

□ measurement of Hba1c

□ measurement of a random glycaemie

□ measurement of glucosuria

□ glucose challenge test:

□ 50g with a cutoff of 130mg/dl

□ 50g with a cutoff of 140mg/dl

□ other: …

□ immediately an OGTT:

□ 2-h 75g

□ 3-h 100g

*Which diagnostic criteria do you use with a 75g OGTT?

□ 2 abnormal values: fasting ≥95, 1-h ≥180, 2-h ≥155

□ 1 abnormal value: fasting ≥126, 2-h ≥140

□ 1 abnormal value: fasting ≥92, 1-h ≥180, 2-h ≥153

* Which diagnostic criteria do you use with a 100g OGTT?

□ 2 abnormal values: fasting ≥95, 1-h ≥180, 2-h ≥155, 3-h ≥140

□ 2 abnormal values: fasting ≥105, 1-h ≥190, 2-h ≥165, 3-h ≥145

**Screening for gestational diabetes ≥ 24 weeks of pregnancy:**

*Do you screen for GDM ≥24 weeks of pregnancy? □ yes □ no

If you screen, what is the estimated percentage of women who receive screening ≥24 weeks of pregnancy?

□ I know… % □ I estimate …% □ I do not know

* If you screen for GDM ≥24 weeks of pregnancy, is this based on:

□ risk factors □ universally

* If you screen based on risk factors, which risk factors do you use? …

* From how many weeks till how many weeks do you generally screen for GDM?...

*Which of the following screening tests do you use:

□ measurement of a fasting plasma glucose

□ measurement of Hba1c

□ measurement of a random glycaemie

□ measurement of glucosuria

□ glucose challenge test:

□ 50g with a cutoff of 130mg/dl

□ 50g with a cutoff of 140mg/dl

□ other: …

□ immediately an OGTT:

□ 2-h 75g

□ 3-h 100g

*Which diagnostic criteria do you use with a 75g OGTT?

□ 2 abnormal values: fasting ≥95, 1-h ≥180, 2-h ≥155

□ 1 abnormal value: fasting ≥126, 2-h ≥140

□ 1 abnormal value: fasting ≥92, 1-h ≥180, 2-h ≥153

* Which diagnostic criteria do you use with a 100g OGTT?

□ 2 abnormal values: fasting ≥95, 1-h ≥180, 2-h ≥155, 3-h ≥140

□ 2 abnormal values: fasting ≥105, 1-h ≥190, 2-h ≥165, 3-h ≥145

**The policy concerning gestational diabetes at delivery**

*Dou you have a protocol for GDM at delivery concerning:

□ monitoring blood glucose

□ the need of an insulin sliding scale

□ the need for an induction

□ the need for a caesarean section

□ neonatal care on the monitoring of blood glucose in newborns

□ the need for admission on the neonatal intensive care unit

**The long term policy after delivery concerning the risk to develop type 2 diabetes**

*Dou you have a protocol concerning the long term follow up of the risk of women with previous GDM to develop type 2 diabetes after the delivery? □ yes □ no

* the follow up strategy includes the following:

□ Monitoring of blood glucose in hospital after the delivery

□ self-monitoring of blood glucose at home

□ a 75g OGTT only in insulin treated women

□ an universal 75g OGTT postpartum

□ fasting plasma glucose

□ HbA1c

□ random glycaemia

□ project ‘Zoet zwanger’

*When an OGTT is performed postpartum, this is:

□ <6 weeks postpartum

□ between 6-12 weken postpartum

□ > 12 weeks postpartum

□ other:…

*The advice given to women with previous GDM, includes:

□ diet and weight control

□ physical activity

□ the frequency and manner of sceening for type 2 diabetes after delivery

□ the need for preconception control when planning a new pregnancy

□ advice on the preferred choice of contraceptives

□other: …
